# Identification of a flavonoid isolated from plum (*Prunus domestica*) as a potent inhibitor of Hepatitis C virus entry

**DOI:** 10.1038/s41598-017-04358-5

**Published:** 2017-06-21

**Authors:** Mihika Bose, Mohini Kamra, Ranajoy Mullick, Santanu Bhattacharya, Saumitra Das, Anjali A. Karande

**Affiliations:** 1 Department of Biochemistry, Indian Institute of Science, Bangalore, 560012 India; 2 Department of Organic Chemistry, Indian Institute of Science, Bangalore, 560012 India; 3 Department of Microbiology and Cell Biology, Indian Institute of Science, Bangalore, 560012 India; 4 Director’s Research Unit, Indian Association for the Cultivation of Science, Kolkata, 700 032 India

## Abstract

Hepatitis C virus (HCV) infection is a major cause of chronic liver diseases that often requires liver transplantation. The standard therapies are limited by severe side effects, resistance development, high expense and in a substantial proportion of cases, fail to clear the infection which bespeak the need for development of well-tolerated antivirals. Since most of the drug development strategies target the replication stage of viral lifecycle, the identification of entry inhibitors might be crucial especially in case of liver-transplant recipients. In the present study we have evaluated fruits which are known for their hepatoprotective effects in order to screen for entry inhibitors. We report the identification of a flavonoid, rutin, isolated from *Prunus domestica* as a new HCV entry inhibitor. Characterization and confirmation of the chemical structure was done by LC-ESI-MS, NMR and IR spectral analyses. Rutin significantly inhibited HCV-LP binding to hepatoma cells and inhibited cell-culture derived HCV (HCVcc) entry into hepatoma cells. Importantly, rutin was found to be non-toxic to hepatoma cells. Furthermore, rutin inhibits the early entry stage of HCV lifecycle possibly by directly acting on the viral particle. In conclusion, rutin is a promising candidate for development of anti-HCV therapeutics in the management of HCV infection.

## Introduction

Hepatitis C virus (HCV) is a leading cause of chronic viral hepatitis that is estimated to infect ~160 million people globally^1^. Persistent HCV infection leads to liver fibrosis, liver cirrhosis and hepatocellular carcinoma^2^. A preventive vaccine against HCV infection is not available. The standard treatment for HCV infection includes the administration of pegylated interferon alpha in combination with ribavirin^3^. However, limitation to this therapy includes low sustained virological response (SVR). In the last few years, treatment options and efficiency have improved with the advent of several classes of direct-acting antivirals (DAAs) that comprise of protease inhibitors, NS5A and NS5B inhibitors. Although the currently approved DAAs have dramatically increased the SVR rates and revolutionized the treatment regimen, they are associated with severe side effects and this therapy may not be able to abrogate the infection in a substantial number of cases^4^. Due to the high genetic heterogeneity and rapid replication of HCV, there is a high risk for development of drug resistant virus strains^5^. Moreover, the high cost associated with this treatment makes it inaccessible to patients in the low resource countries. Therefore, there is an imperative need for the development of new antivirals that are well tolerated, less expensive and more readily available. Despite the fact that most of the DAAs target the replication step of HCV lifecycle, recent studies have depicted that addition of entry inhibitors to the DAAs exert a synergistic effect on the efficiency of antiviral treatment^6^. Therefore, combination of inhibitors targeting different stages of the virus lifecycle including entry, replication and assembly/secretion might be a better therapeutic strategy to reduce the risk of viral escape mutants. Also, chronic infection is associated with end-stage liver disease which represents the major cause of liver transplantation^7^. In majority of the patients, HCV re-infection is seen to occur in the grafted liver^8^. Donor allograft reinfection can be prevented by inhibiting viral entry into hepatocytes using entry inhibitors.

Genotype 3a of HCV is most prevalent in India, therefore we initiated our studies using hepatitis C virus-like particles (HCV-LPs) comprising of core-E1-E2 derived from genotype 3a. The study was carried out using HCV-LP based system since virus-like particles (VLPs) mimic the morphology of native virus and represent a well-established system for studies on viral binding and entry^9, 10^.

For decades, traditional medicines have been used for the cure of various diseases. An extensive variety of phytochemicals are demonstrated to possess antiviral activity. Compounds from natural sources have been reported for their antiviral effect against various infections such as herpes simplex virus^11^, influenza virus, human immunodeficiency virus (HIV)^12, 13^ hepatitis B and even hepatitis C virus^14, 15^.

The fruits and vegetables explored for anti-HCV activity in this study were chosen based on their hepatoprotective effect. Of the several extracts examined, plum (*Prunus domestica*) exhibited the highest inhibitory activity. The isolation, identification, structural elucidation and anti-HCV properties of the compound rutin (a flavonoid) are presented in this paper. We evaluated the efficiency of rutin in blocking the binding and entry of HCV-LPs in hepatoma cells and also assessed the potential of rutin in the prevention of HCV Japanese fulminant hepatitis 1 (JFH1) infection *ex vivo*.

## Results

### Evaluation of toxicity of the different extracts

Fruits and vegetables that are known to possess hepatoprotective property were selected as mentioned in Table 1. To investigate the cytotoxic effect of these extracts, Huh 7 cells were treated with different concentrations of the extracts and cell viability was assessed by MTT [3-(4,5-dimethylthiazol-2-yl)-2,5-diphenyltetrazolium bromide] assay. Extracts of the fruits plum, apple, black grape, red grape, cranberry, blackberry, tomato, jujube and that of the vegetable, beetroot (Supplementary Fig. S1) did not exhibit significant cytotoxicity. Extracts of papaya and dates displayed cytotoxicity at higher concentrations (Supplementary Fig. S1). Garlic extract was found to have significant toxicity even at low concentration and was not investigated further (Supplementary Fig. S1).

**Table 1:** List of fruits and vegetables used for the study.

Scientific Name	Common Name	Plant part used
*Prunus domestica*	Plum	Fruit
*Malus domestica*	Apple	Fruit
*Vitis vinifera*	Black Grape	Fruit
*Vitis labrusca*	Red Grape	Fruit
*Vaccinium oxycoccos*	Cranberry	Fruit
*Rubus fruticosus*	Blackberry	Fruit
*Solanum lycopersicum*	Tomato	Fruit
*Carica papaya*	Papaya	Fruit
*Ziziphus jujuba*	Jujube	Fruit
*Phoenix dactylifera*	Dates	Fruit
*Beta vulgaris*	Beetroot	Taproot
*Allium sativum*	Garlic	Bulb

### Inhibition of HCV-LP binding to hepatoma cells by extracts

**Figure 1:**
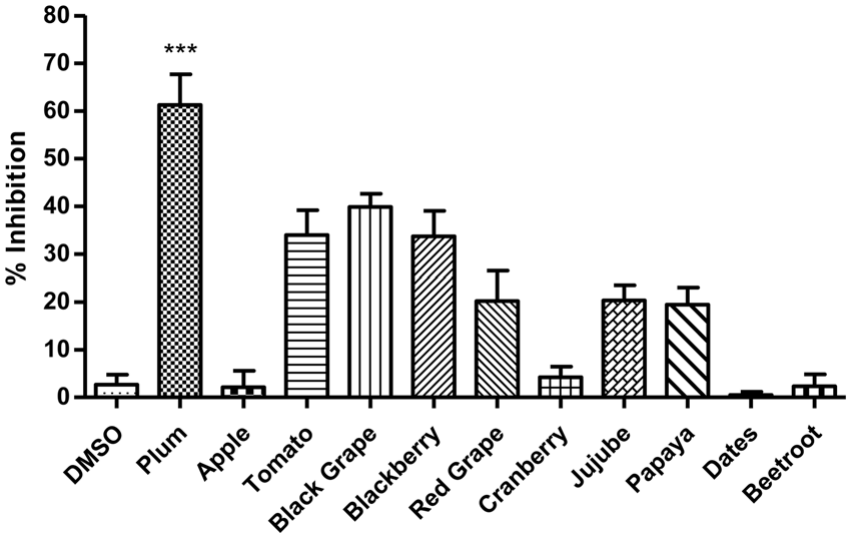
Screening of different extracts for inhibition of HCV-LP binding to hepatoma cells. Huh 7 cells were incubated with the extracts (200 μg/ml) along with Alexa-488 labelled HCV-LPs for 2 h at RT. The binding of labeled HCV-LPs was determined by flow cytometry considering DMSO as negative control. The x axis represents the different extracts and the y axis represents the percentage inhibition of HCV-LP binding. The graph represents data pooled from at least three independent experiments and each sample in duplicates. Error bars represent the standard deviation.

To evaluate the inhibitory activity of the extracts on the binding of HCV to Huh 7 cells, a screening assay was carried out using Alexa-488 labelled HCV-LPs. Cells were incubated with HCV-LPs in the presence of the different extracts (200 μg/ml) and inhibition of binding was determined by flow cytometry. The extract of plum exhibited highest inhibition (~62%; *P* < 0.0001) of HCV-LP binding to Huh 7 cells compared to the extracts of tomato, blackgrape and blackberry that exhibited moderate inhibition (Fig. 1). The remaining extracts did not manifest any significant inhibition. Since the plum extract exhibited the highest activity, it was selected for further studies to identify the bioactive compound responsible for its anti-HCV activity.

### Isolation of the bioactive component with antiviral activity against HCV

**Figure 2:**
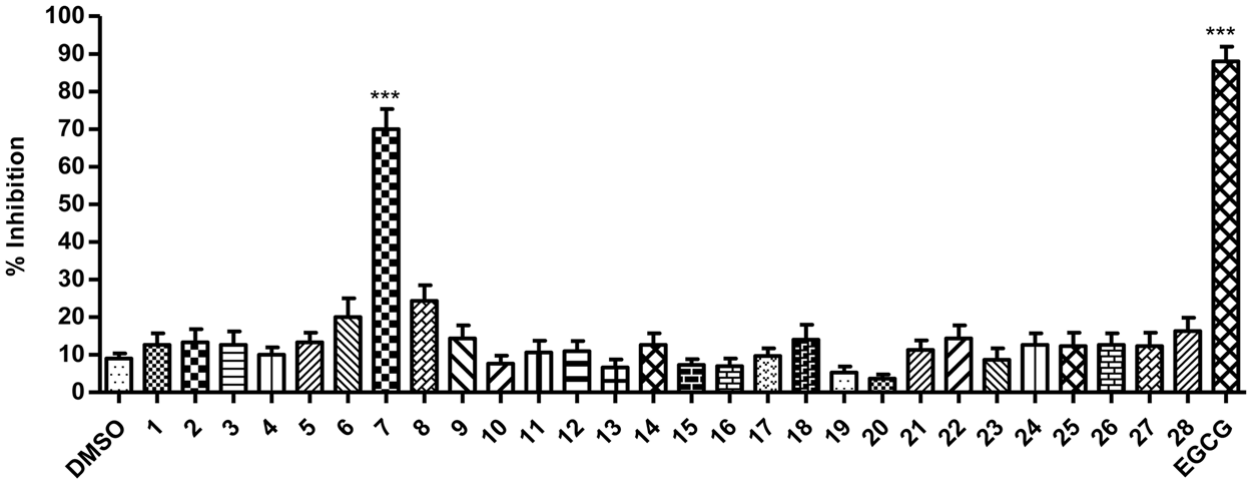
Screening of TLC derived fractions for antiviral activity. Alexa-488 labelled HCV-LPs were incubated with Huh 7 cells and 20 μg/ml of the TLC-separated fractions (from the extract of *Prunus domestica)*, for 2 h at RT. The binding of HCV-LPs was assessed by flow cytometry. DMSO and EGCG were taken as negative and positive controls respectively. The x axis represents the different fractions and the y axis represents the percentage inhibition of HCV-LP binding. All data are pooled from at least three independent experiments and the standard deviation is represented as error bars.

The crude extract of plum (*Prunus domestica*) was fractionated by thin layer chromatography (TLC). The extract was subjected to analytical preparative TLC-guided fractionation in the selected solvent system (chloroform: methanol: acetonitrile). The resulting 28 fractions were further assessed for inhibition of HCV-LP binding to Huh 7 cells. Alexa-488 labelled HCV-LPs were incubated with Huh 7 cells along with each of these components or EGCG [(-) epigallocatechin-3-gallate], a known HCV entry inhibitor^16, 17^ at a concentration of 20 μg/ml and binding of VLP to hepatoma cells was monitored by flow cytometric analysis. Only fraction 7 [Retention factor (R_f_) value 0.2] showed significant inhibition (~70%; *P* < 0.0001) of VLP binding to hepatoma cells (Fig. 2).

### Identification of the bioactive compound with antiviral activity

**Figure 3:**
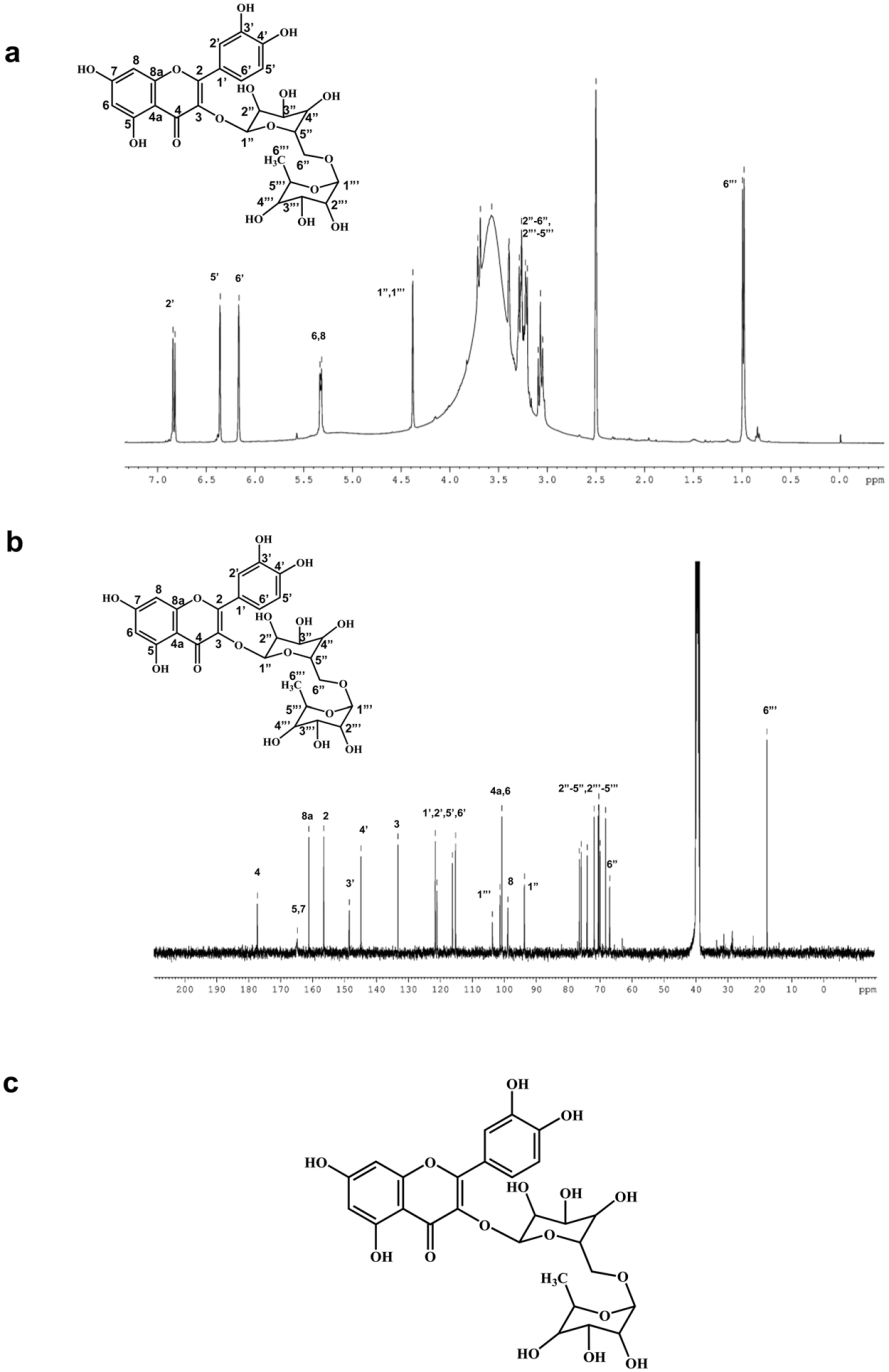
Identification of the bioactive compound with antiviral activity against HCV. (**a**,**b**) ^1^H and ^13^C nuclear magnetic resonance (NMR) spectroscopic data of the fraction 7. The compound rutin was identified according to the NMR spectroscopy ^1^H (**a**) and ^13^C (**b**) and chemical shifts are referenced to the solvent signal (^1^H NMR, δ 2.5 ppm; ^13^C NMR, δ 39.51 ppm). (**c**) Chemical structure of rutin (drawn using ChemDraw Ultra 8.0).

The chemical characterization of fraction 7 was accomplished next. A single prominent peak was observed in the high performance liquid chromatography (HPLC) profile, suggesting the presence of a single compound corresponding to fraction 7 with more than 90% purity (>90% pure) (Supplementary Fig. S2). Liquid chromatography-electrospray ionization- mass spectrometry (LC-ESI-MS) studies were carried out to ascertain the molecular weight of the compound constituting fraction 7. In the positive ion mode, the compound spectrum displayed the presence of the molecular ion peak (m/z = 611.1565; [M+H]^+^ = 611.1606) as depicted in Supplementary Fig. S3. Based on this, the constituents of plum reported in literature, were noted and for the identification of the molecular structure among those, nuclear magnetic resonance (NMR) studies of the purified compound 7 were conducted. Both the ^1^H and ^13^C NMR spectra of the compound point towards the flavonoid, rutin (Fig. 3a and b). In order to confirm the structure we also performed spectral analyses with the commercially available rutin-hydrate and both the ^1^H and ^13^C NMR spectra of the commercial rutin-hydrate (Supplementary Fig. S4) were in complete agreement with those of the isolated compound. The Fourier transform infra-red (FT-IR) spectrum also confirmed the presence of C-O of methyl ether, cyclic ether and glycosidic linkage (1020, 1049 and 1090 cm^−1^) and O-H of alcohol (3300–3400 cm^−1^) as functional groups, thereby supporting the structure of rutin (Supplementary Fig. S5). Elemental analyses were performed for both, the isolated and commercial compounds. The analysis of the commercially available rutin hydrate (Sigma) gave the % composition, C 48.86, H 5.06, O 46.08. These values lie close to those calculated for rutin trihydrate (C_27_H_36_O_19_), C 48.80, H 5.46, O 45.74. Analysis of the isolated compound pointed to the hexahydrate form of rutin (C_27_H_42_O_22_) calcd: C 45.13, H 5.89, O 48.98; found: C 44.60, H 5.81, O 49.59. This may be attributed to the hygroscopic nature of the compound, rutin. Our results demonstrated that the flavonoid, rutin (Fig. 3c) is responsible for the anti-HCV property of *Prunus domestica*.

### Effect of rutin on HCV-LP binding to hepatoma cells

**Figure 4:**
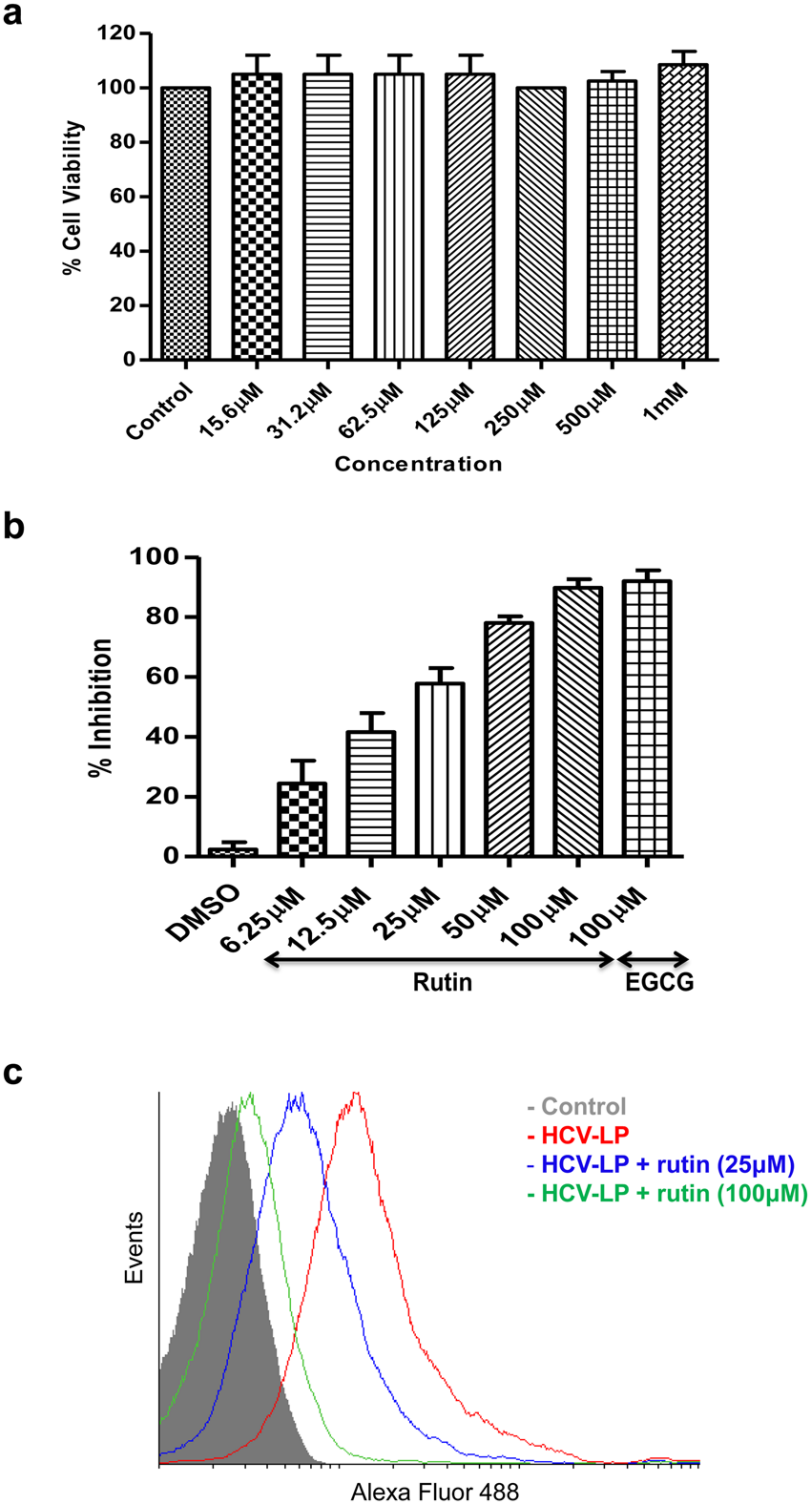
Characterization of rutin. (**a**) Evaluation of cytotoxicity of rutin. Increasing concentrations of the compound was incubated with Huh 7 cells for 48 h after which the cell viability was measured by MTT assay. The x-axis represents concentrations of rutin and the y-axis represents the percentage cell viability. (**b**,**c**) **Inhibition of HCV-LP binding to hepatoma cells by rutin**. (**b**) Huh 7 cells were incubated with Alexa488-labelled HCV-LPs along with increasing concentrations of rutin. The cellular binding of HCV-LPs was monitored by flow cytometry. The x axis represents the different concentration of rutin and y axis represents the percentage inhibition of HCV-LP binding. DMSO and EGCG were considered as negative and positive controls respectively. The assays were performed at least three times. (**c**) Histogram representation of rutin-mediated inhibition of HCV-LP binding to hepatoma cells. Huh 7 cells alone were represented as control.

We first examined the cytotoxic effect of rutin on the host cells before evaluating its antiviral activity against HCV. For this purpose cell viability was determined by MTT cell proliferation assay. It is clearly demonstrated from the results that rutin is not toxic to hepatoma cells even at 1 mM concentration exposed for 48 h (Fig. 4a). To evaluate the potential antiviral activity of the isolated flavonoid, rutin, increasing concentrations of the compound was incubated simultaneously with Alexa-488 labelled HCV-LPs and Huh 7 cells. Flow cytometric analysis was performed to measure the reduction of binding of VLPs to Huh 7 cells. Our data indicates a significant inhibition of binding in the presence of rutin in a dose dependent manner (Fig. 4b and c). The half maximal inhibitory concentration (IC_50_) of rutin was estimated to lie between 12.5 µM–25 µM concentrations and the 90% inhibitory concentration (IC_90_) dose was ~100 µM.

### Mechanism of rutin-mediated inhibition

**Figure 5:**
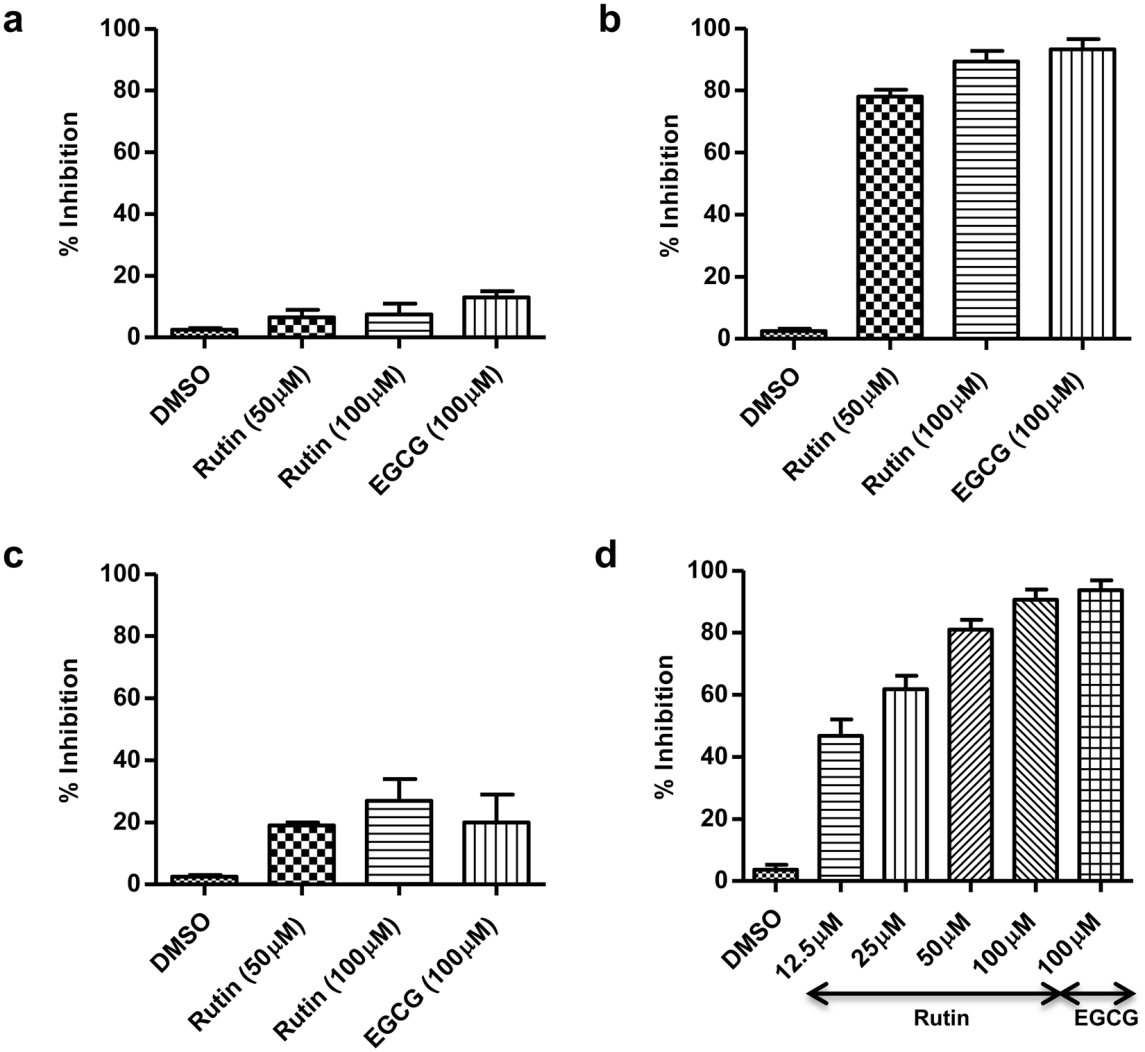
Rutin inhibits HCV binding and entry by acting on the viral particle. Rutin was added before (**a)**, during (**b**) or after (**c**) the addition of Alexa488-labelled HCV-LPs to Huh 7 cells. After 2 h of incubation cell bound fluorescence was assessed by flow cytometry. (**d**) Labelled HCV-LPs were pre-incubated with rutin followed by addition of Huh 7 cells. The binding of HCV-LPs was evaluated by flow cytometry. DMSO and EGCG serve as negative and positive controls respectively. The x axis represents the compounds and the y axis represents the percentage inhibition of VLP binding. All data are pooled from three independent experiments performed in duplicates.

HCV entry is a multistep process and to characterize the step inhibited by rutin, time of addition experiment was carried out wherein the compound was administered at different time windows, before, during or after addition of labelled HCV-LPs to Huh 7 cells. There was a significant inhibition of HCV-LP binding to hepatoma cells only in the presence of rutin (~78% and ~90% at concentrations of 50 µM and 100 µM respectively) (Fig. 5b) whereas there was no effect of rutin if added at a post-attachment step (Fig. 5c) or if added to cells prior to the addition of VLP (Fig. 5a). Therefore, our data suggests that rutin might inhibit the attachment step of virus entry thereby blocking at the initial phase.

Since rutin did not manifest any considerable inhibition if added to cells before exposure to the VLPs (pre-treatment of cells), it does not act on the target cells but might act on the viral particles. When labelled HCV-LPs were pre-incubated with rutin and then added to Huh 7 cells, there was a significant inhibition of fluorescence associated with the cells (Fig. 5d). Thus, it can be concluded that rutin inhibits HCV-LP binding to Huh 7 cells by directly acting on the viral particle.

**Figure 6:**
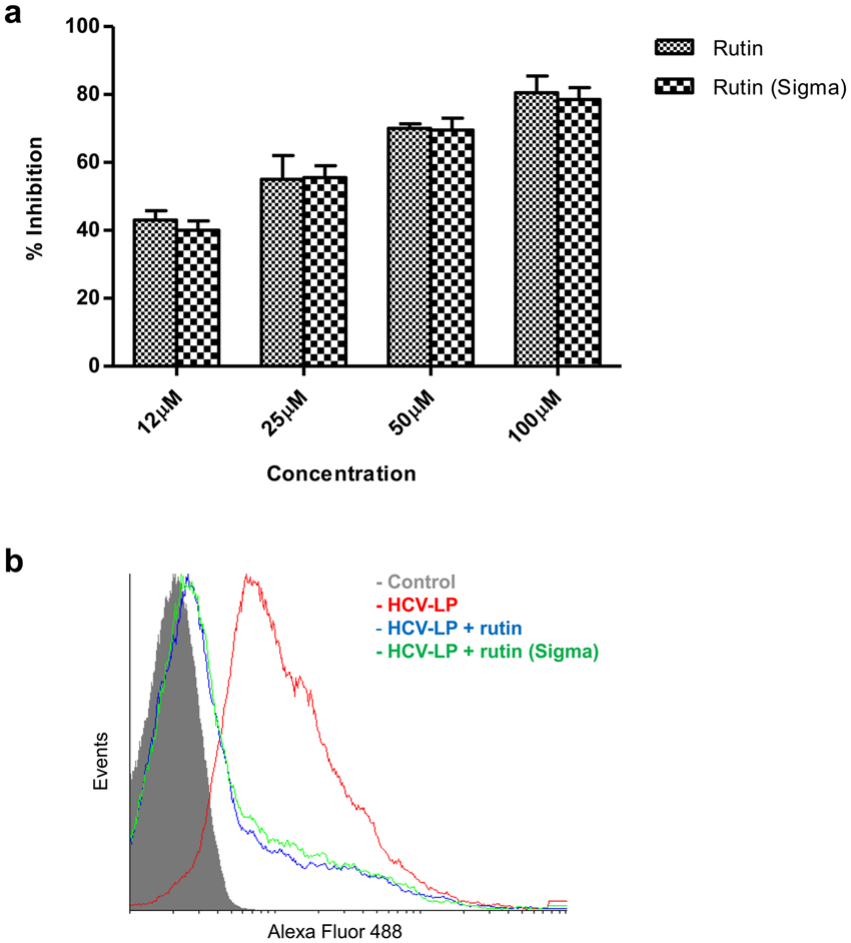
Comparison of inhibitory activity of purified and commercially available rutin. (**a**) Alexa488- labelled HCV-LPs was incubated with Huh 7 cells along with increasing concentrations of purified rutin or commercial rutin-hydrate. The cellular binding of HCV-LPs was measured by flow cytometry. The x axis represents the different concentration of the compounds and y axis represents the percentage inhibition of HCV-LP binding. (**b**) Histogram representation of inhibition of HCV-LP binding to hepatoma cells by rutin and rutin-hydrate. Huh 7 cells alone were taken as control. The graphs represent pooled data from at least three independent experiments.

For the final confirmation of the inhibitory effect of rutin, the antiviral property of the isolated rutin was compared with the commercially available rutin. Results show that the inhibitory activity of the rutin purified from plum was comparable with the commercially available rutin and the half maximal inhibitory concentration for both of them was estimated to be between 12.5 µM and 25 µM (Fig. 6a and b).

### Rutin inhibits HCVcc entry and infection

**Figure 7:**
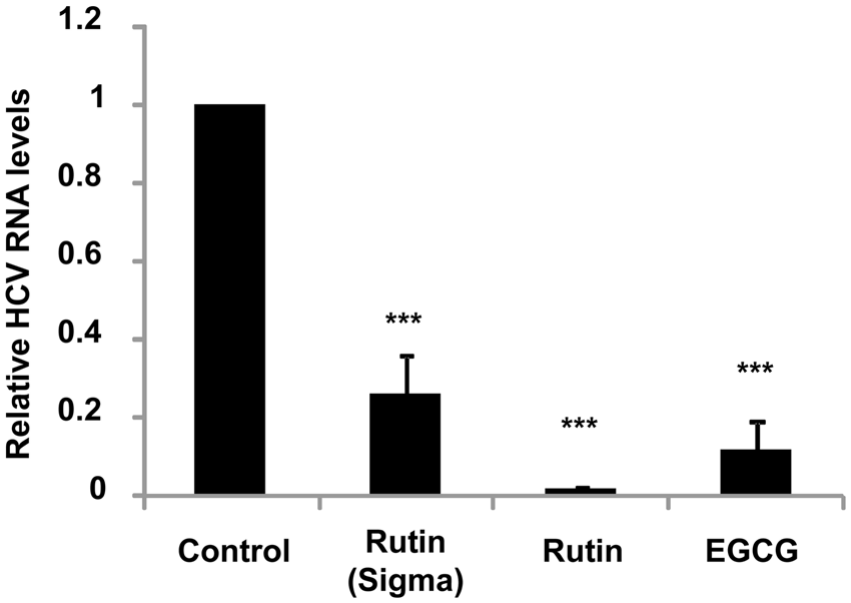
Rutin mediated inhibition of HCV entry and infection in cell culture. JFH1 virus was preincubated with or without 100 µM of rutin or rutin-hydrate at 37 °C followed by infection of Huh 7.5 cells for 4 h. Total cellular RNA was isolated three days post-infection and using real time PCR, HCV negative strand was quantified. A TLC fraction of plum extract that did not inhibit HCV-LP binding to cells *in vitro* is represented as control and EGCG is considered as positive control.

To investigate the inhibitory effect of rutin in the context of the virus we evaluated the effect of rutin on HCV entry in the cell culture system (HCVcc) where virions of hepatitis C are used. The infectious JFH1 virus was preincubated with the purified compounds and infected Huh 7.5 cells at 37 °C for 4 h. HCV negative strand synthesis was quantified by real time PCR (polymerase chain reaction) three days post infection. Inhibition of HCV entry by rutin was determined indirectly by the measurement of decrease in the level of intracellular HCV RNA. A significant reduction in the intracellular HCV RNA level in the presence of both rutin and commercially available rutin was observed (>95% and ~80% respectively; *P* < 0.0002) compared to the control fraction that did not inhibit *in vitro* (Fig. 7).The above observation confirms the antiviral effect of rutin in the cell culture system (*ex vivo*).

## Discussion

Various compounds isolated from plants are known to harbour activity against several viral infections including HCV. The potential use of these natural compounds is highlighted since they are safe and less expensive. The recent trend is inclined towards the development of biologically active drugs from natural sources, that can be easily metabolised and absorbed by the body, as is evident by the continued discovery and development of herbal medicines of new formulations^14^. Since they do not exhibit any side effects, these compounds might supplement or circumvent the present treatment regimen. Even if natural compounds might not replace the present treatment regimen for HCV infection, they could be considered adjunct therapy. Though in most instances, the replication step of the HCV life cycle is targeted, entry inhibitors like ladanein, silymarin and EGCG have been observed to be potential antivirals against HCV^15^. Recently, the flavonoid delphinidin has been added to the list of HCV entry inhibitors^18^. However, entry inhibitors are still in the stage of preclinical trials^19^.

HCV entry is a well-orchestrated process that involves interaction of the envelope glycoproteins with several cell surface receptors. For effective antiviral therapy, the entry inhibitors have great potential since they target the initial phase of infection. Moreover, most of the DAAs target the replication stage of HCV infection; therefore, identification of entry inhibitors is essential for combination therapy to target the various stages of HCV life cycle for better therapeutic outcome. In our quest for identifying natural compounds (as entry inhibitors) for anti-HCV therapy, we evaluated the effect of extracts from different fruits and vegetables that are known to harbour hepatoprotective property. Among the several extracts tested, the extract of plum (*Prunus domestica*) significantly inhibited (~62%) binding of HCV-LPs to hepatoma cells whereas the extracts of tomato, blackgrape and blackberry showed moderate inhibitory activity. Since the extract of plum exhibited highest inhibition, we selected it for further studies. Moreover, cell viability assay revealed that the extract of plum was not toxic to hepatoma cells.

Due to the high content of antioxidants and polyphenolics, dietary intake of fruits has a wholesome approach for the cure of various diseases. *Prunus domestica*, commonly known as plum belongs to the family *Rosaceae*. This fruit is appreciated by consumers all around the world and is familiar for laxative and stomachic effect. Plums are rich in anti-oxidants^20, 21^ and phytochemical studies indicate the presence of flavonoids, flavonoid glycosides, abscisic acid, lignans, carotenoid pigments, quinic acid, bipyrole and dihydroflavonols^20, 22, 23^.

In this study, we purified the bioactive compound responsible for the anti-HCV activity of plum. Structural elucidation studies identified the compound to be rutin, which was characterised and confirmed by LC-ESI-MS, NMR and FT-IR spectral analysis. The isolated compound belongs to the flavonoid family. We demonstrated rutin as a new inhibitor of HCV entry. Flow cytometric analysis revealed that rutin significantly inhibited binding of labelled HCV-LPs to Huh 7 cells. The half maximal inhibitory concentration (IC_50_) was estimated to lie between 12.5 µM and 25 µM concentration and the IC_90_ dose was close to 100 µM which is similar to the reported active concentration of other flavonoids (10–200 μM)^17^. Rutin was not toxic to hepatocytes even after 48 h of treatment at high concentrations (upto 1 mM). Time of addition experiment suggest that rutin probably inhibits the attachment of HCV to host cell surface thereby interfering at the early entry step. Rutin failed to inhibit HCV-LP binding to hepatoma cells when the cells were pre-treated with the compound whereas significant inhibition was observed when the viral particles were treated suggesting that rutin directly acts on the viral particle. Therefore, it might be hypothesised that rutin targets the envelope glycoproteins thereby preventing virus-receptor interaction. That the active principal of plum extract is indeed rutin was further confirmed by comparing the plum purified rutin with that of the commercially available rutin-hydrate. Flow cytometric analysis revealed that both rutin and rutin-hydrate exhibit comparable inhibitory activity. Rutin also inhibited HCV entry and infection in the HCVcc system (*ex vivo*) significantly (>95%) which is revealed by the marked reduction in the HCV negative strand synthesis in real time PCR. Taken together, these results demonstrate that rutin inhibits HCV entry both *in vitro* as well as *ex vivo*.

In case of HCV patients undergoing orthotropic liver transplantation, re-infection of the donor allograft is a common phenomenon^7, 8^. Prevention of graft re-infection is a major clinical goal that can be achieved by means of preventing HCV entry into hepatocytes thereby reinforcing the potential use of entry inhibitors like rutin. Multi-drug therapy with a combination of drugs targeting the different stages of the life cycle of HCV would be more efficacious in the management of the viral disease. Thus, entry inhibitors can be used along with DAAs as an effective therapeutic strategy.

Studies by Zuo *et al*. have reported the activity of rutin against the NS3 serine protease, *in vitro* ^24^. Rutin is a 3-*O*-glycoside derivative of quercetin; the latter is also a known NS3 protease inhibitor^25^. We speculate that the presence of the sugar moiety, rutinose, in rutin might mask the relevant residues on the viral surface thereby interfering with virus-receptor interaction, although further analysis of the intricate mechanism is required. Due to its dual function, rutin might be a lead structure for future drug development in anti-HCV therapeutics. It is also reported that quercetin glycosides like rutin are partially deglycosylated in the small intestine and are not affected by the pH of the stomach^26^. Apart from its anti-inflammatory property^27^, rutin is also known to protect liver cells by its antioxidant activity^28^.

In conclusion, detailed spectral analysis based on ^1^H NMR and proton-decoupled ^13^C NMR of the bioactive fraction and commercially available rutin leads us to the identification of rutin as a new HCV entry inhibitor. Further chemical characterization of the compound confirms the structure of this flavonoid, quercetin 3-*O*-rutinoside. The compound was found to significantly inhibit viral entry and infection in both, HCV-LP (*in vitro*) and HCV cell culture (*ex vivo*) systems. Herein, we report rutin, a component of plum that can have potential use as an antiviral agent in the prevention and management of HCV infection.

## Methods

### Cells

Human hepatocellular carcinoma cells Huh 7 and Huh 7.5 (gift from Dr. Charles M. Rice, Apath LLC, St.Louis, MO)^29^ were cultured in Dulbecco’s modified Eagle’s medium (DMEM, Sigma-Aldrich), supplemented with 10% foetal bovine serum (FBS), glutamax (Invitrogen) and antibiotics (100 u/ml penicillin, 100 µg/ml streptomycin and 5 u/ml nystatin). The cultures were maintained in a humidified incubator under 5% CO_2_ at 37 °C. The Sf21 cells were cultured in TC100 (Sigma) insect cell medium supplemented with 10% FBS, at 26–28 °C.

### Purification of HCV-LPs derived from genotype 3a

The gene corresponding to core-E1-E2 of genotype 3a (Acc. No. core: GU172376 and E1E2: GU172375) was cloned in baculovirus expression system and the recombinant baculovirus was generated as described previously^30^. Sf21 cells were infected with the recombinant virus at a moi of 5–10 and 72 h post infection the cells were harvested. Cell pellets were subsequently washed with PBS (50 mM phosphate buffer, pH 7.2 containing 150 mM NaCl), resuspended in lysis buffer (50 mM Tris, 50 mM NaCl, 0.5 mM ethylenediaminetetra-acetic acid (EDTA), 1 mM phenylmethylsulfonyl fluoride (PMSF), 0.1% NP40 and 0.25% protease inhibitors) and homogenized. The lysate was centrifuged at 4 °C at 10,000 rpm for 15 min and the resulting supernatant was pelleted over a 30% sucrose cushion. Following this, the pellet was resuspended in 20 mM Tris, pH 7containing 150 mM NaCl and was applied on a 20–60% continuous sucrose gradient for ultracentrifugation in SW41 rotor (Beckman and Coulter). Ultracentrifugation was performed at 30,000 rpm for 22 h at 4 °C. The fractions (1 ml) collected were tested for presence of HCV-LPs. HCV-LP containing fractions were diluted with PBS and then pelleted at 30,000 rpm for 2 h. The pellet was dissolved in PBS followed by storage at −80 °C for future use.

### Labelling of HCV-LPs

HCV-LPs were labelled with Alexa-488 dye (Invitrogen) as described earlier^31, 32^. Briefly, the VLPs (2 mg/ml) were dissolved in 1 ml of 0.1 M sodium bicarbonate buffer, pH 9 to which 10 μl of 10 mg/ml Alexa dye was added followed by incubation at room temperature (RT) for 2 h with constant stirring. The labelled VLPs were separated from the free dye on a Sephadex G-25 desalting column.

### Preparation of extracts

All the fruits and vegetables used in this study were bought from the local market and were sliced and then pulverised in a grinder. 50 g of each sample was extracted with 100 ml of ethyl acetate for 24 h at RT under constant shaking. The extracts were filtered to remove insoluble debris and were concentrated under reduced pressure using Rotary Evaporator (IKA, Germany). Next, the Slurry was air-dried and stored at −20 °C for future use.

### Purification and identification of the bioactive compound

Thin layer chromatography (TLC) was performed for the isolation of the active component from the extract of plum (*Prunus domestica*). For purification, preparative TLC was carried out in the selected solvent system of chloroform: methanol: acetonitrile in the ratio of 8:1:0.1 using precoated silica gel 60 F254 plates (0.5 mm; 20 × 20 cm) (Merck, Germany). Crude extract of plum (50 mg) was resuspended in a minimal volume of HPLC grade ethyl acetate and spotted on silica gel (20 × 20 cm), dried and developed using the selected solvent system as the mobile phase. The chromatogram was observed under a UV lamp at wavelengths of 254 nm and 366 nm and for each band the retention factor (R_f_) was determined. Each band was scraped and extracted using HPLC grade methanol and using PTFE (polytetrafluoroethylene) filter (0.22 μm) all the fractions were filter sterilized. Fraction 7 (that showed anti-HCV activity) was subjected to HPLC using a reverse-phase column (Thermo Finnigen surveyor C_18_ column, 3 µm, 4.6 × 150 mm) and isocratic method of elution was performed with methanol: water (70: 30) with a flow rate of 1 ml/min. For identification and characterization of the compound, we performed LC-ESI-MS [(LC: Dionex Ultimate 3000 using a reverse phase column C_18_, 5 µm, 4.6 × 150 mm, gradient elution with 10–90% acetonitrile+ 0.1% formic acid − water+ 0.1% formic acid with a flow rate of 200 µl/min) and MS: Q-TOF Impact HD (Bruker)], ^1^H NMR (Bruker Avance 400 at 400 MHz), ^13^C NMR (Bruker Avance 400 at 100 MHz) and FT-IR spectral analyses (Bruker ALPHA). All chemical structures were drawn and analysed using ChemDraw Ultra 8.0.

### Preparation of samples for *in vitro* and cell culture studies

Stocks of the different crude extracts were prepared in DMSO at a concentration of 100 mg/ml (w/v) and subsequently diluted in serum free DMEM for the different assays. Stocks of the purified compounds, rutin, EGCG (Sigma) and rutin hydrate (Sigma) were dissolved at a concentration of 15 mM in DMSO and diluted in DMEM before use.

### Assessment of cytotoxicity of the extracts

MTT [3-(4,5-dimethylthiazol-2-yl)-2,5-diphenyltetrazolium bromide] assay was performed using standard protocol^33^, to determine the cytotoxic effects of extracts/compounds. Huh 7 cells (5 × 10^3^) were plated in 96-well plates for 24 h in DMEM at 37 °C followed by addition of varying concentrations of the extracts (final concentration ranging from 30 μg/ml to 1 mg/ml), or the purified compound (final concentration ranging from 15.6 μM to 1 mM). The plates were incubated in the presence of the extracts for 24 h or in the presence of the purified compound for 24 h and 48 h. Next, 20 μl of MTT (Sigma-Aldrich) solution was added to each well followed by incubation at 37 °C for 4 h. The MTT solution was carefully aspirated and in each well DMSO (100 µl) was added for the extraction of the formazan crystals from cells. The colour intensity was measured at 550 nm (reference filter set to 620 nm) using a microplate reader (VERSA max tunable, Molecular Devices).

### Inhibition of HCV-LP binding to Huh 7 cells by extracts/purified compounds

Huh 7 cells (4 × 10^4^) were incubated with Alexa-488 labelled HCV-LPs (20 μg/ml) either in the absence or presence of the different crude extracts (at a final concentration of 200 μg/ml) or different fractions obtained from TLC (at a final concentration of 20 μg/ml) or varying concentrations of purified compounds [rutin or rutin-hydrate (Sigma, Aldrich)] at RT for 2 h followed by subsequent washes with DMEM (serum-free) to remove unbound VLPs. Using flow cytometry (FACS Canto II flow cytometer; Becton Dickinson) the cell bound fluorescence was analysed with CYFLOGIC^TM^ software (CyFlo Ltd, Turku, Finland) to calculate the mean fluorescence intensity (MFI) of the cell population which directly relates to the surface density of the Huh 7 cells bound labelled HCV-LPs^30, 32, 34^. The % binding of HCV-LP was determined as [experimental MFI- control (only cells) MFI/Positive control MFI- control (only cells) MFI] × 100. EGCG at the same concentration and DMSO have been used as positive and negative controls respectively.

### Time of addition experiment

Rutin was added at different time points, either before, during or after addition of Alexa 488-labelled HCV-LPs to Huh 7 cells. For pre-treatment of cells, rutin was added to the cells, and incubated for 1 h, washed with DMEM (serum free) followed by addition of labelled HCV-LPs. After incubation for 2 h the cell bound fluorescence was measured by flow cytometry as previously described. For post-attachment assay, cells were incubated with labelled HCV-LPs for 1 h and then rutin was added and further incubated for 2 h. As demonstrated above, cell bound fluorescence was measured by flow cytometry.

In case of pre-treatment of VLPs, rutin was pre-incubated with the labelled HCV-LPs for 1 h followed by addition of the complex to Huh 7 cells and incubated for 2 h. The remaining procedure is similar as above. EGCG and DMSO were used as positive and negative controls respectively.

### Neutralisation of virus (JFH1) in cell culture

The cell culture derived infectious HCV-JFH1 viral particles were generated as described previously^29, 35^. The intracellular HCV RNA level was measured to determine the ability of the compounds (rutin, commercial rutin and EGCG) to inhibit HCV entry. The JFH1 viral particles were preincubated with the purified compounds for 1 h at 37 °C followed by infection of the virus-compound complex in Huh 7.5 cells and incubated for 4 h at 37 °C. A TLC-derived fraction of plum extract that did not show inhibition *in vitro* was used as control and EGCG was used as positive control. Post infection cells were washed twice with PBS and changed to complete medium. Infectivity was analysed 3 days post-infection by real-time PCR. The total RNA was isolated and reverse- transcribed using HCV IRES 5′ forward primer and GAPDH 3′reverse primer in order to detect HCV negative strand and using SYBR green PCR master mix (Thermo Scientific), the resulting cDNA was quantified. The ABI ViiA7 real time PCR system was used to amplify the cDNA for HCV IRES and GAPDH (internal control). The set of primers used for the experiment are as follows:

HCV 5′forward primer: 5′-TGCGGAACCGGTGAGTACA-3′

HCV 3′reverse primer: 5′-CTTAAGGTTTAGGATTCGTGCTCAT-3′

GAPDH 5′forward primer: 5′-CATGAGAAGTATGACAACAGCCT-3′

GAPDH 3′reverse primer: 5′-AGTCCTTCCACGATACCAAAGT-3′

### Statistical analysis

All data were analysed using GraphPad Prism5 software. Unpaired *t*-test was performed for the determination of statistical significance. Data represent mean ± standard deviation from at least three independent experiments performed in duplicates.

## Electronic supplementary material

Supplementary information
