# Supplementary material for: Identification of a flavonoid isolated from plum (Prunus domestica) as a potent inhibitor of Hepatitis C virus entry
Source: Sci Rep. 2017 Jun 21;7:3965. doi: 10.1038/s41598-017-04358-5 (PMC5479801; doi:10.1038/s41598-017-04358-5)
Supplement: Supplementary file 1 — Supplementary information [file 41598_2017_4358_MOESM1_ESM.pdf]

Supplementary Information

for

Identification of a flavonoid isolated from plum (*Prunus domestica*) as a potent inhibitor of Hepatitis C virus entry

Mihika Bose[1], Mohini Kamra[2], Ranajoy Mullick[3], Santanu Bhattacharya[2, 4], Saumitra Das[3] and Anjali A. Karande[1*]

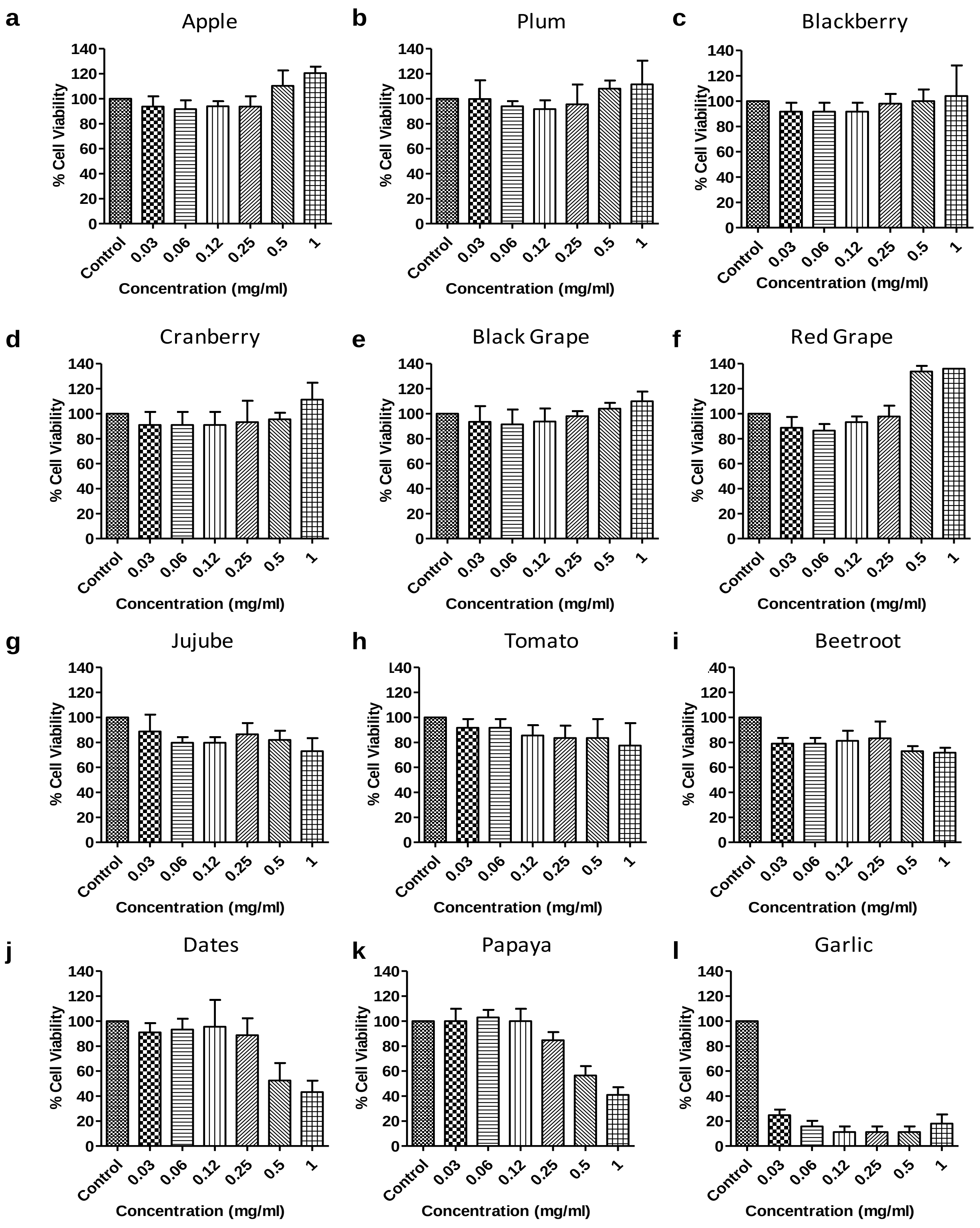

Supplementary Fig. S1: Evaluation of cytotoxic effect of the extracts: Increasing concentrations of the different extracts were incubated with Huh 7 cells for 24 h after which the cell viability was measured by MTT assay. The x-axis represents concentrations of the extracts and the y-axis represents the percentage cell viability considering 100% for the solvent control (DMSO).

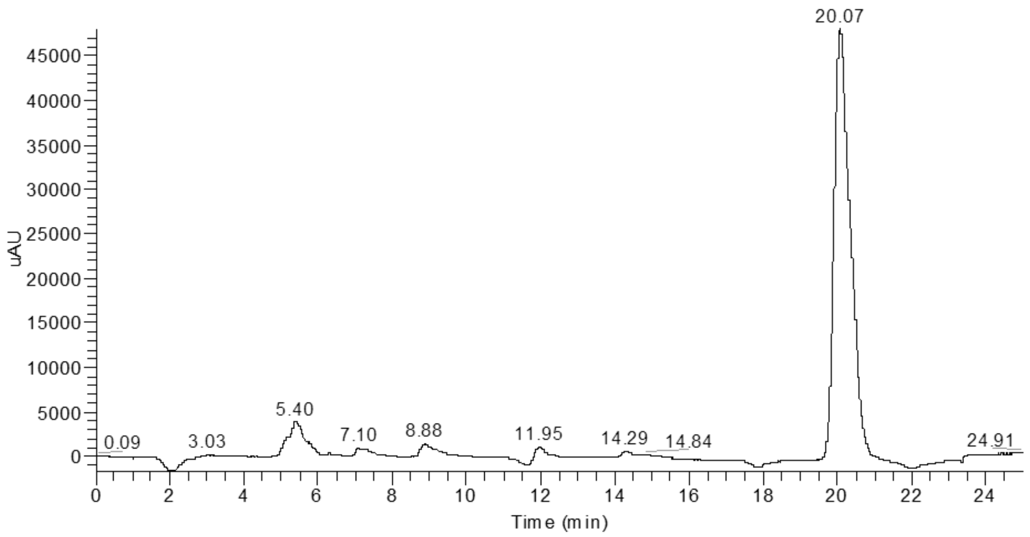

Supplementary Fig. S2: HPLC profile of fraction 7: HPLC chromatogram of rutin depicts a prominent major peak.

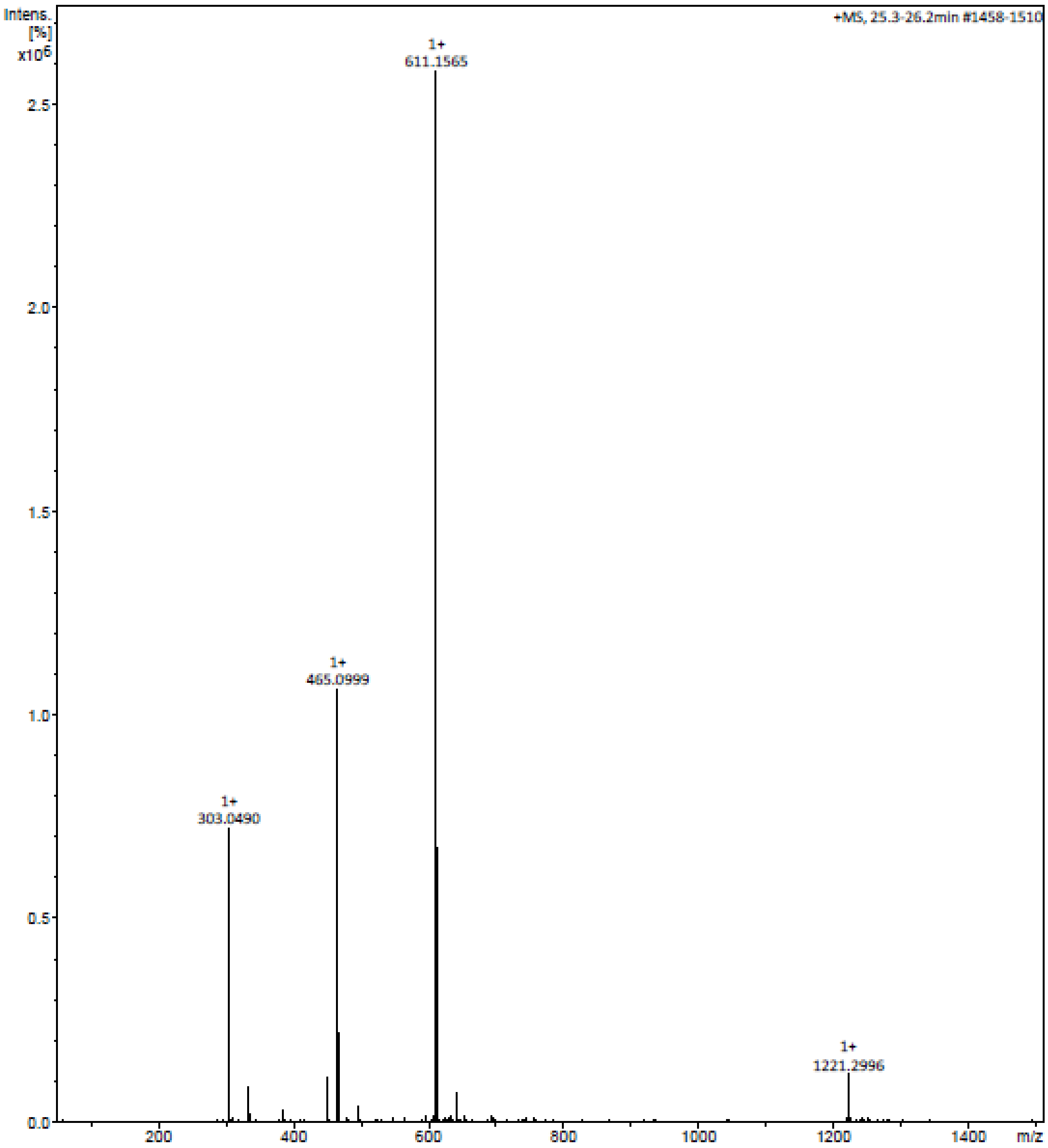

Supplementary Fig. S3: High resolution mass spectrum of rutin isolated from plum extract: LCMS of the purified compound shows the molecular ion peak (m/z = 611.1565; $[M+H]^+$ = 611.1606) in the positive ion mode.

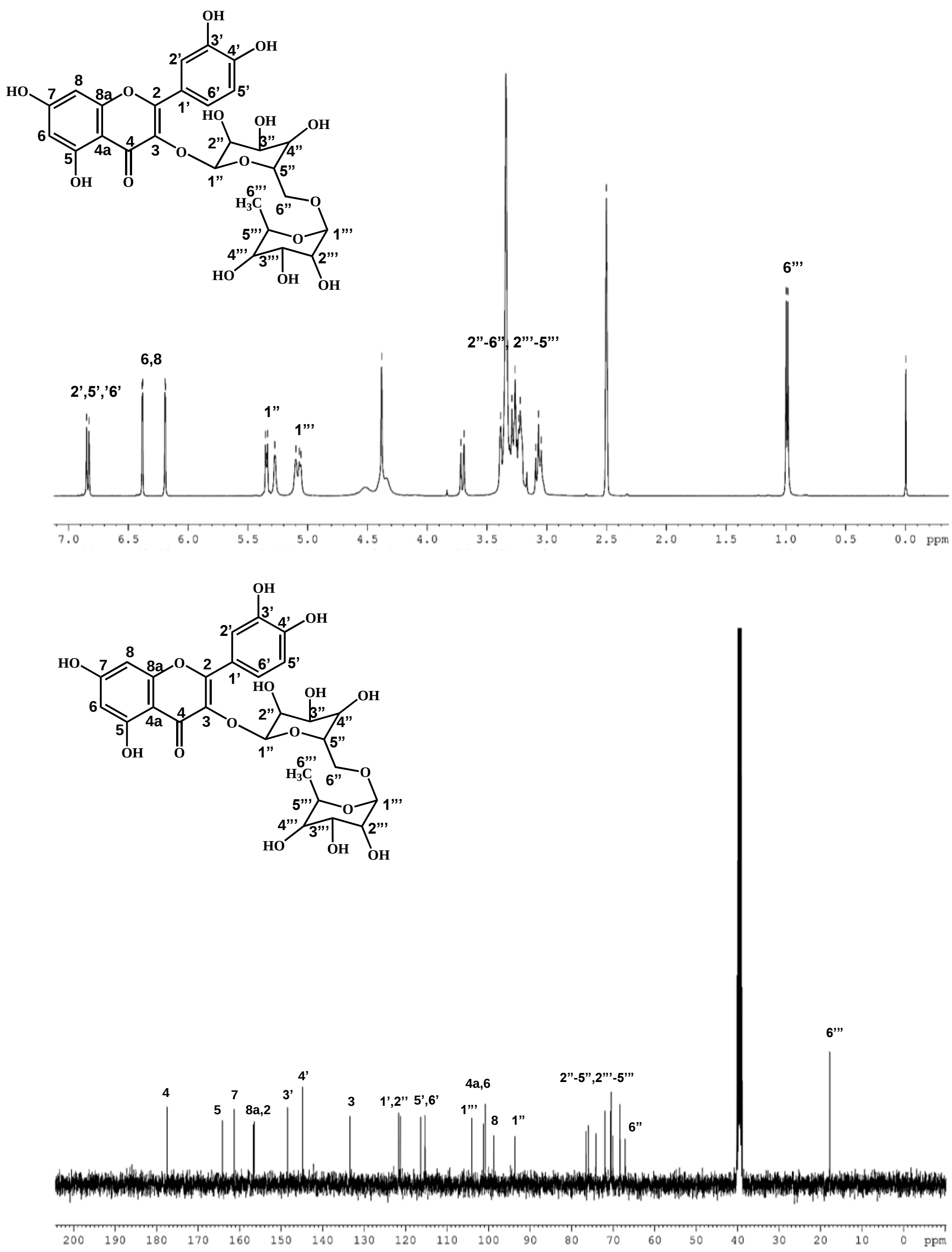

Supplementary Fig. S4: ^{1}H and ^{13}C nuclear magnetic resonance (NMR) spectroscopic data of rutin-hydrate: The NMR spectroscopy of the compound; ^{1}H (top) and ^{13}C (bottom). Chemical shifts are referenced to the solvent signal (^{1}H NMR, δ 2.5 ppm; ^{13}C NMR, δ 39.51 ppm).

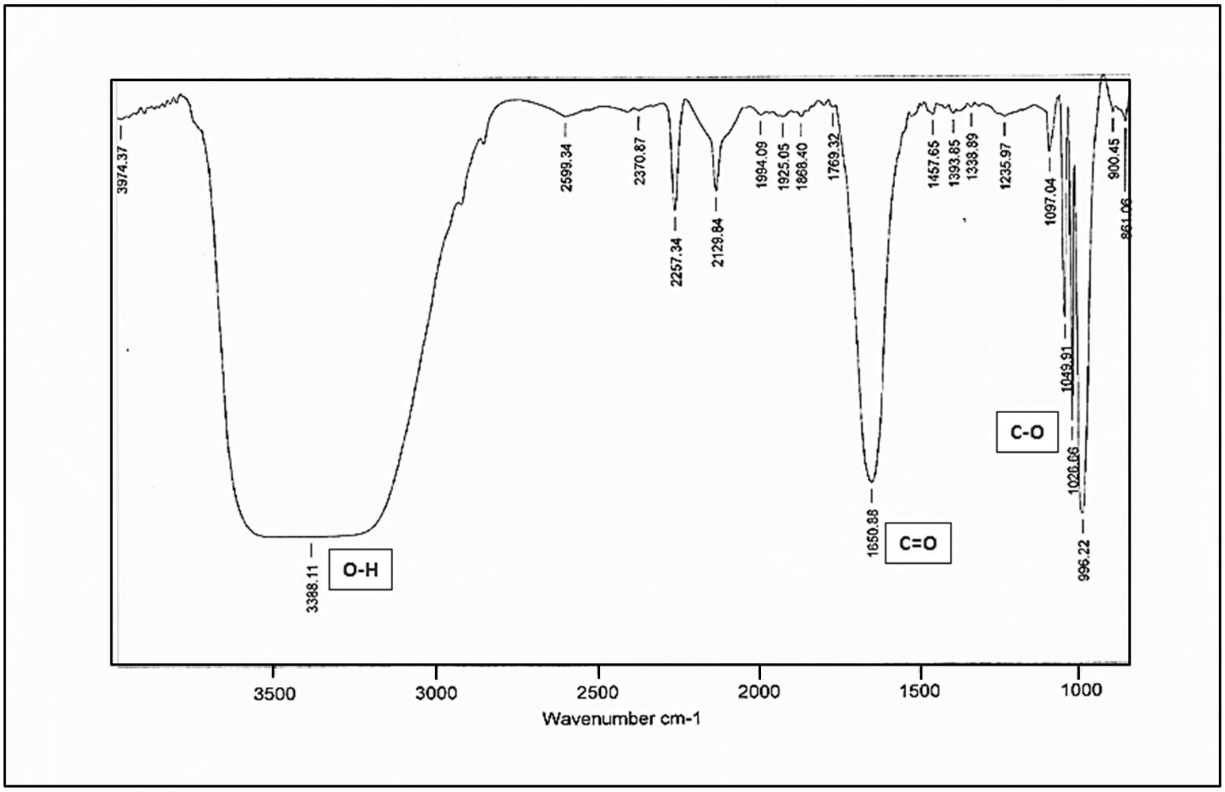

Supplementary Fig. S5: Infra-Red spectrum of rutin: The IR spectrum represents the presence of C-O of methyl ether, cyclic ether and glycosidic linkage (1020, 1049 and 1090 cm^{-1}) and O-H of alcohol (3300-3400cm^{-1}) as functional groups.
